# A micro-credentialing methodology for improved recognition of HE employability skills

**DOI:** 10.1186/s41239-021-00315-5

**Published:** 2022-02-23

**Authors:** Marcelo Fabián Maina, Lourdes Guàrdia Ortiz, Federica Mancini, Montserrat Martinez Melo

**Affiliations:** Universitat Oberta de Catalunya, Rambla de Poblenou 156, 08020 Barcelona, Spain

**Keywords:** Micro-credentialing methodology, Employability skills, Social integrative pedagogy, ePortfolio, Micro-credential, Badge, Skill articulation, Higher education, Skills recognition, Employers

## Abstract

Increasingly, among international organizations concerned with unemployment rates and industry demands, there is an emphasis on the need to improve graduates’ employability skills and the transparency of mechanisms for their recognition. This research presents the Employability Skills Micro-credentialing (ESMC) methodology, designed under the EPICA Horizon 2020 (H2020) project and tested at three East African universities, and shows how it fosters pedagogical innovation and promotes employability skills integration and visibility. The methodology, supported by a competency-based ePortfolio and a digital micro-credentialing system, was evaluated using a mixed-method design, combining descriptive statistics and qualitative content analysis to capture complementary stakeholder perspectives. The study involved the participation of 13 lecturers, 169 students, and 24 employers. The results indicate that the ESMC methodology is a promising approach for supporting students in their transition from academia to the workplace. The implementation of the methodology and the involvement of employers entails rethinking educational practices and academic curricula to embed employability skills. It enables all actors to broaden their understanding of the relationship between higher education and the business sector and to sustain visibility, transparency, and reliability of the recognition process. These findings indicate that there are favourable conditions in the region for the adoption of the approach, which is a meaningful solution for the stakeholder community to address the skills gap.

## Introduction

The need to improve the quality and relevance of skills development and their visibility and comparability for better career perspectives is emphasized in “Agenda 2063” (African Union Commission, 2015). Likewise, the International Labour Organization (ILO) highlights the positive impact of skills recognition on the labour market, particularly with regard to matching skills and jobs (Braňka, 2016). The Organization for Economic Co-operation and Development Skills Strategy (OECD, 2011) continues to acknowledge the importance of skills and their recognition (OECD, 2021). In this scenario, higher education institutions (HEIs) are increasingly focusing on their students’ employability skills as an integral part of their goals (Suleman, 2018) to improve their chances on entering the economic and labour market.

In parallel, the shift of focus from the recognition of conventional qualifications to micro-credentials has also emerged as a trend (Kato et al., 2020). The special interest in their adoption stems from a long-standing debate on the value of degrees for the future of work (Gallagher, 2018). As a consequence, one of the current challenges for education policies and systems is to provide students with the option to accumulate meaningful, skills-focused digital credentials in order to meet today’s workforce requirements.

Micro-credentials offer an opportunity to bridge the skills gap that is acknowledged to make the transition from post-secondary education to the world of work difficult and which is affecting many developing countries, some of which belong to the East African Community (ILO, 2020). Against this background, a twofold strategy is required to respond to the growing concern among employers about the preparation of African graduates for the workplace (Leopold et al., 2017). On one hand, HEIs in Africa need to start questioning how to reduce this discrepancy by designing and implementing curriculum innovation and supporting platforms where employability skills can be recognised and easily shared, thus enhancing the visibility of student achievements. On the other hand, industry partners need to participate in innovations in traditional modes of recognition (Guàrdia et al., 2021) in order to deliver effective solutions that aid in the selection of the best-qualified graduates for employment.

## Purpose of the study

This article presents the methodological approach designed in the EPICA H2020 project for micro-credentialing employability skills of students approaching graduation which is supported by (a) a competency-based ePortfolio as a transition tool from academia to the workplace along with (b) a digital micro-credentialing system addressed to make the skills visible to prospective employers. Additionally, the article reports on the results of a pilot that took place at three East African universities: Maseno University (Kenya), Makerere University (Uganda), and the Open University of Tanzania (Tanzania). The evaluation of the methodology considers the complementary perspectives of the three different stakeholder groups directly involved in the process: lecturers, students and employers. The guiding research questions of the study were:

- RQ1. Which pedagogical innovations focused on employability skills integration and recognition could be fostered by the Employability Skills Micro-credentialing (ESMC) methodology?
- RQ2. How may the ESMC methodology promote employability skills visibility, transparency and trustability for employability purposes?

The focus of the study is on the academic community, particularly on lecturers and students’ perspectives regarding the capacity of the methodology to foster curriculum innovation toward micro-credentialing for employability skills and, hence, to enhance employment opportunities. Special attention was paid to key actors in the professional context and their need to optimise candidate recruitment processes, and the analysis therefore also draws on the employer perspectives regarding the pertinence of an approach that encourages students to showcase badges as evidence of their proficiency in specific skills.

## Background

### Context and the significance of the problem

The lack of focus in HEIs on helping students develop the skills they need to be fully equipped to enter the labour market is one of the reasons for the skills gap reported in the East-Africa region (Njeg’ere Kabita & Ji, 2017). Similarly, the African Development Bank (2019) emphasizes the relevance of adopting a skills-based approach to learning to enhance the employability of undergraduates.

Despite the description of the skills and competencies associated with a particular qualification provided by both the National Qualifications Frameworks (NQFs) and the East African Qualifications Framework for Higher Education (EAQFHE), traditional credentials are proving to be insufficient to address the growing disconnect between what employers want and what the credential communicates (Nikusekela et al., 2016). Credentials, in fact, leave out what and how students learned, and the skills and competencies they acquired within and beyond the walls of the university (Wienhausen & Elias, 2017) which makes them inadequate to reflect the transferable skills needed in a changing workplace. As a consequence, they are becoming increasingly ineffective as a screening mechanism for recruiters.

The need to include more granular skills, abilities and dispositions in class-level learning outcomes also led Transforming Employability for Social Change in East Africa (TESCEA) to undertake different initiatives with the aim of transforming HE for employability and social change (Wild & Omingo, 2020). Among these initiatives, a TESCEA partnership joined forces to develop a skills matrix for graduates’ skills and employment to guide the course redesign process within the university partners. This growing emphasis on the identification of graduate employability skills, suggests micro-credentials as a possible solution to the challenges facing the African labour market today.

### Micro-credentials as a contribution to enhance employability

The European Commission (2020) defines a micro-credential as a certified document issued by an institution or organization of learning outcomes achieved through a learning experience, following quality assurance standards, and containing additional information regarding the holder’s name, the applied assessment methods, and, “where applicable, the qualifications framework level and the credits gained” (p.5). They are owned by the recipient who can share them, combine them with others, or showcase them in different digital contexts. According to Milligan and Kennedy (2017), micro-credentials are part of “a digital credentialing ecosystem, made possible by digital communications technologies establishing networks of interest through which people can share information about what a learner knows and can do” (p.43).

As reported by Oliver (2019), micro-credentials can stand alone or interact with formal qualifications. In the latter case, they may be used as an alternative entry mechanism to degree programs, a means to provide value-added programs during degrees or/and a way to connect ready-to-graduate learners to work experience and employment. Micro-credentials when used to add value to academic programs, therefore, make it possible to acknowledge skills and experiences that are not shown on academic transcripts or CVs, including interpersonal skills and extracurricular or volunteer activities (Braxton et al., 2019) and hence complement conventional qualifications and map career paths.

To sum up, micro-credentials enable the capturing of the extensive range of experiences and skills that students develop during their careers and showcase them to employers as additional signals that go beyond traditional transcripts. The trust of relevant stakeholders in the skills certified by micro-credentials, however, should be fostered by a digital credentialization system that makes relevant information behind the micro-credential (contents, quality, outcomes, assessment, workload, etc.) digitally available and presents it appropriately. The provision of transparent information on the learning experience that led to the credential also addresses the perennial problem that arises from the different ways HE and the labour market describe achievements (Orr et al., 2020).

### Micro-credentialing employability skills in the higher education curriculum

HEIs are one of the key providers of micro-credentials and well placed to drive innovation in this area (MICROBOL, 2020). They have a crucial role in guiding students in the aggregation of learning experiences into structured learning journeys and in recognising the skills required for success in the workplace (Gauthier, 2020).

Integrating and micro-credentialing skills in HE curricula, however, requires that academic institutions overcome the knowledge transfer paradigm in favour of active learning models and authentic assessment scenarios (Sokhanvar et al., 2021) that surface both academic knowledge and workplace skills (Kilsby & Goode, 2019).

Additionally, institutions should take into account not only the employability skills students learn throughout the curriculum but also those developed outside the classroom. Capturing evidence of learning gained in different contexts in a way that both supplemental learning achievements and non-cognitive attributes are visible (Tyton Partners, 2015) requires institutions to reframe the way outcomes and learning are recognised.

According to Selingo (2017), students’ ePortfolios of assets and data enhance the credibility of micro-credentials by documenting the learning process, progress and performances. In this way, employers are able to see the incremental advances that students have made and use this information in hiring decisions. Additionally, the fact that ePortfolios are integrative in nature promotes a process of reflection and articulation by the students of the range of experiences, knowledge, and competencies that constitute their education (Wienhausen & Elias, 2017). Digital badges are often paired with ePortfolios. Although the terms digital badges and micro-credentials have been often used interchangeably in the literature, digital badges usually make reference to the process through which a technological system handles and issues them. Digital badges, therefore, should be intended as “a representation of an accomplishment, interest or affiliation that is visual, available online, and contains metadata that help explain the context, meaning, process and result of an activity” (Gibson et al., 2015, p.403). These electronic symbols stored in the competency-based ePortfolio provide recognition of students’ skills, and give access to a collection of evidence about their capacities and readiness for work. The intersection between digital badges and ePortfolios, when successful, can unlock the power of the evidence behind the badge and enhance both the learner’s ability to present a collection of projects and their capacity to make claims about their competences (Ambrose et al., 2016). The pairing of badges and ePortfolios has also been identified as a future direction for research and practice (Eynon & Gambino, 2017).

### The EPICA micro-credentialing methodology

With the aim of providing graduates with more detailed accounts of their learning and accrediting their employability skills, the EPICA project developed and tested a new methodology for employability skills assessment, micro-credentialing and visibility that employs a competency-based ePortfolio as a transition tool, where evidence is attached to badges (Maina et al., 2020). The methodology draws from different successful initiatives. First, Deakin University’s Professional Practice Credentials (Jorre De St Jorre et al., 2016) which adopts the use of evidence, rubrics and micro-credentials to certify professional skills acquired throughout a career. Second, the UWaterloo curriculum vitae project (WatCV) that supports students in articulating their skills focusing on their transferability to the workplace, and the use of a digital portfolio as a high-impact educational practice (Watson et al., 2016), the Catalyst Framework (Eynon & Gambino, 2017), and the STAR (Situation, Task, Action, Results) method, a structured manner of answering behavioural interview questions focusing on skills and the strategic description of significant lived experiences, which is frequently used in hiring processes. Third, the Comprehensive Learner Record project (Green & Parnell, 2017) which highlights the importance of developing a record showing achievements in employability skills to complement the HE degree and transcript, and linking them with evidence coming from curricular and extracurricular experience. Finally, the VALUE initiative (McConnell et al., 2019) on students learning outcomes assessment, provided a set of initial generic rubrics for adaptation and application.

**Fig. 1:**
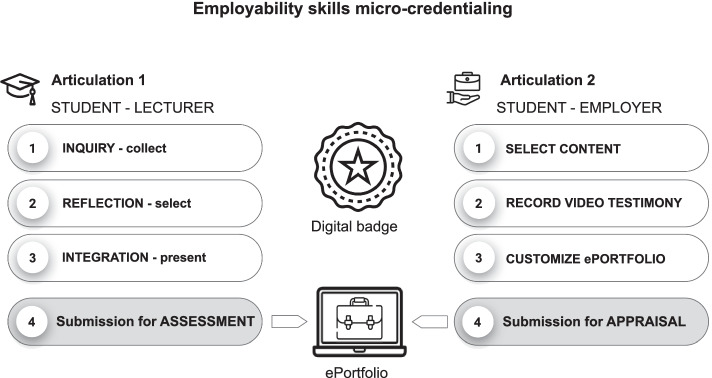
EPICA methodology—Micro-credential process overview (Source: Authors. EPICA Initiative)

The underlying assumption for the design of this methodology is that university courses and programs currently do provide opportunities for the development of employability skills but they are not directly and explicitly managed or assessed (Tomasson Goodwin et al., 2019). This premise led to the identification of a solution capable of raising awareness of the significant skills already addressed, albeit unintentionally, by the curriculum and engaging students, lecturers, and employers in a collaborative endeavour focused on micro-credentialing these skills. This process was organised in two articulation phases (see Fig. 1) in which the students developed the ability to identify and communicate their skills to specific target groups (Guàrdia et al., 2021).

#### Articulation 1

In the first articulation, students were required to articulate their employability skills in written, visual and verbal form for lecturers by engaging in inquiry, reflection and integration tasks following the pedagogical design principles of the Catalyst Framework (Eynon & Gambino, 2017) and using an ePortfolio. Hence, they had to:

- Inquire into their own curricular and extracurricular experiences to identify significant situations within and outside their academic contexts which could provide direct or indirect evidence to demonstrate the development of their employability skills;
- Reflect on how the situations they had identified contributed to the development of these skills and select pieces of evidence that best illustrate their application. Each piece of evidence is presented with a description providing contextual explanation;
- Integrate their learning by elaborating a reflective narrative that carefully explains and justifies how the overall evidence presented demonstrates their skills. A skill-specific rubric with the assessment criteria enables students to self-assess their level of development, and thus guide the justification and the presentation of the evidence.

Lecturers scaffold their students throughout the whole process providing formative feedback and encouraging the submission of their work. The ability to demonstrate the selected employability skills is then assessed by academics on the basis of a customized rubric. Effective showcasing is acknowledged by a digital badge per skill showcased.

#### Articulation 2

The second articulation of the micro-credential process is aimed at ensuring that students are able to effectively communicate their employability skills to employers. Students are required to review their profiles and personalize their ePortfolios by:

- Writing or reviewing the presentation of themselves, including their short bios and pictures and other relevant information considered of interest to an employer;
- Reviewing their awarded digital badges and evidence to start developing a script of a short video presentation;
- Adding new evidence on achievements that contribute to build a more comprehensive portrait of their capacities;
- Recording a 3 to 5-min video testimony to communicate their profile to a prospective employer following the STAR method and highlighting their experiences and achievements;
- Customize the ePortfolio paying attention to the formal organization of all elements and to aesthetic aspects before sharing with an employer for appraisal.

Employers are required to appraise the employability skills on the basis of the student’s presentation and the evidence provided through the competency-based ePortfolio. In cases where the showcased work is of exceptional quality, employers may endorse the student with a written personal commendation.

Both articulations are bridged by the digital badge as the main evidence that serves as formal academic recognition linked to experiences and achievements and thus providing substantive information on the student’s capacities.

## Materials and methods

### Research context

The pilot of the EPICA ESMC methodology took place between January and July 2020 and, despite the COVID outbreak that forced it to be done completely online, it involved the participation of 13 lecturers, 169 students, and 24 employers. The micro-credentialing process was implemented, taking as a reference Ornellas et al. (2019) employability skills taxonomy, in 11 bachelor programs from different disciplines including Education, Law, Management, Mathematics, Social Work, Nursing, Informatics, and others. During this experience the 169 students engaged in the process of assessment of 2 to 4 employability skills each, accounting for a total of 526 assessed skills, mainly creative thinking (161, 30.6% of skills), communication and interpersonal skills (139, 26.4%), and problem-solving (108, 20.5%). 136 students (80%) then continued with Articulation two showcasing their badges to potential employers. It is worth mentioning that the employers were expressly appointed to the pilot for research purposes. The future deployment of the methodology is expected to be managed by university staff and integrated into the curriculum as part of an innovation aiming at increasing graduates employability opportunities.

### Research design

A mixed-method approach was applied, combining qualitative and quantitative techniques, and convergent design (Clark & Ivankova, 2016), to capture the perspectives of participating African universities students, lecturers and appointed employers.

**Fig. 2:**
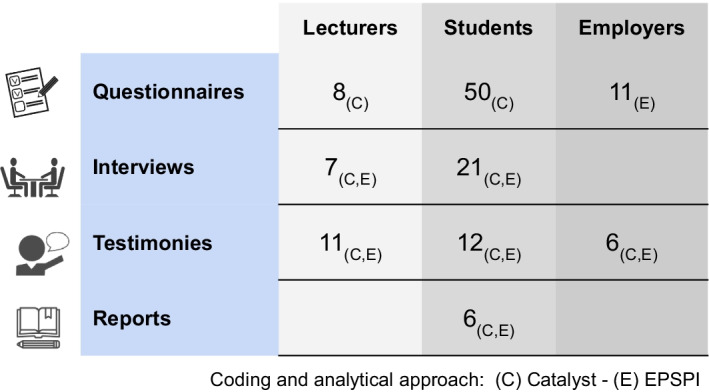
Research instruments and participants (Source: Authors. EPICA Initiative)

A set of data collection instruments was designed (see Fig. 2) based on the Catalyst Framework (Eynon & Gambino, 2017) dimensions: inquiry, reflection, integration, and outcomes assessment. And, with the aim of gaining insights into students and lecturers’ perceptions of the potential of the methodology to enhance employability opportunities, the Electronic Portfolio Student Perspective Instrument, EPSPI (Ritzhaupt et al., 2010) was also integrated into the analysis. EPSPI involves four distinct constructs defined as primary purposes: learning, assessment, visibility, and employment. In particular, the latter two provided information relating to the use and relevance of the EPICA solution in the transition from university to the workplace.

The students and lecturers’ online questionnaire was answered by 50 and 8 participants respectively and measured the Catalyst dimensions on a scale of 1–7. The employers’ questionnaire drew on EPSPI and was answered by 11 African employers. In parallel, 28 semi-structured interviews based on EPSPI were carried out with students (21) and lecturers (7). Additionally, student reports, following a similar script, were produced by six students (2 per university) which described their experience in a narrative form. By the end of the project, 29 free and open testimonies produced by students (12), lecturers (11), and employers (6) were also collected and included in the analysis as a secondary source, as they provided a valuable narrative corpus.

### Data analysis

As part of a pilot project, the group of participants represent a non-probabilistic sampling by criterion where inferential validity and reliability probabilistic measures do not apply. Considering the sample sizes, the quantitative results of students, lecturers, and employers’ questionnaires have been analysed applying univariate descriptive statistics with the SPSS**©** program. The results are reported through parameters appropriate to the variables metrics and sample size.

Qualitative content analysis (Schreier, 2012) was applied to the corpus of data from the students and lecturers’ interviews, students’ reports, and employers’ open questions, as well as testimonies. Atlas.ti**©** was used for coding and analysis. A coding manual (Syed & Nelson, 2015) was developed based on Catalyst and EPSPI.

The code attributes were established in a collaborative exercise among the researchers, including definition and inclusion criteria (Creswell & Poth, 2018). Two researchers individually started by coding 20% of the total data as a trial coding before comparison for consistency. The accuracy and scope of the codes were discussed, and adjustments were made. This process assisted in the refinement of the existing codes and the identification of other emerging ones in a deductive-inductive approach (Elo & Kyngäs, 2008). To reach a consensus on the meaning attributed to data and to ensure the reliability of the results, this procedure was also carried out with the data from students and employers. The codes were clustered into categories (see Table 1, in Appendix), and semantic networks representing visual depictions of the conceptual structure and the connections between concepts (see Figs. 6, 7, 8, in Appendix) were created to increase the understanding of each group of participants’ views.

**Table 1:** Qualitative analysis. Source: Authors

Categories	Lecturers	Students	Employers
Pedagogical principles	-Inquiry, reflection, integration process -Outcomes assessment	-Inquiry, reflection, integration process -Outcomes assessment	-Inquiry, reflection, integration process
Employability enhancement	-Employability -Skills showcase -Transparency -Visibility -Micro-credentials	-Employability -Skills showcase -Transparency -Visibility -Micro-credentials	-Employability -Skills showcase -Micro-credentials -Transparency -Visibility -Bridge the gap -Recruitment
Experience	-ESMC process implementation -Professional development -Students learning gains -Stakeholders engagement	-Learning gains -Platform -Student training -Stakeholders participation	-Appraisal process -Stakeholders engagement -Students learning gains
Methodology Uptake	-Adoption potential -Scalability	-Adoption potential -Scalability -Proposals for future implementation	-Adoption potential

Codes and categories

## Results

This section presents lecturers, students, and employers’ perspectives organized into three research concerns: the pedagogical principles underlying the micro-credentialing process, the micro-credentials and ePortfolio for employability enhancement, and the methodology uptake.

### Lecturer perspective

#### Pedagogical principles

**Fig. 3:**
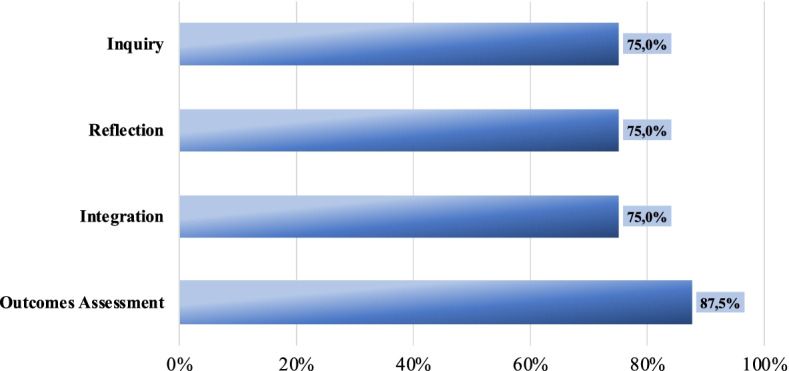
Lecturers’ perceptions of pedagogical principles fostered by the ESMC. Total lecturers: 8. Percentage of agreement based on a 1–7 scale (1 = Strongly disagree and 7 = Strongly agree) (Source: Authors. EPICA Initiative.)

Lecturers evaluated the pedagogical principles for employability skills micro-credentialing (ESMC) positively both in the questionnaires (see Fig. 3) and interviews. The questionnaires revealed promising results with little variations regarding inquiry, reflection, and integrative learning (75%, n = 6 lecturers).

The lecturers highlighted that the methodology enabled students to inquire into their experience and identify appropriate situations and evidence demonstrating the acquisition of skills. The collection of the evidence along with the articulation of skills fostered reflection on their learning journey and contributed to a better understanding of their strengths in relation to the requirements of the labour market. One of the lecturers mentioned that “*the ePortfolio requires students to upload their own evidence where they can actually understand what they’ve learnt, and show to the employers exactly what they know*”. Some others explained that this experience has helped students to “*improve their self-confidence in various fields of study*” and, even though “*there were some challenges, they became more resilient; it was a full win situation*”.

Additionally, lecturers concurred that the methodology provides strong support for outcomes assessment (87.5%, n = 7). Specifically, they stated that the ePortfolio has proven to be a very useful tool for assessing students’ achievements across an entire programme. The use of rubrics was also appreciated as they provide common criteria and detailed descriptors that increase transparency and homogeneous assessment. Moreover, some lecturers highlighted the benefits of formative feedback and continuous assessment, especially in the process of identifying the best evidence in support of their skills demonstration. This brings opportunities to students to fine-tune their submissions. The interaction among students and lecturers was also perceived by the latter as a valuable strategy to support reflection and to improve the awareness of their own employability skills: “through *ePortfolio, students can simply send their activities online and get immediate feedback for further improvement where necessary. Contrary to traditional assessment, students can be assessed several times to make sure that they are able to fully demonstrate their capacity in a particular skill*”.

The qualitative analysis also showed that the implementation of the methodology prompted a conceptual change that influenced the lecturers’ teaching practices as shown in the following quote: “*Prior to my participation in the project, I* did not know how to incorporate employability skills in my lecturing activities. I can now include employability skills in the assignments and tasks. My module design skills have also greatly improved”. Lecturers concurred that this experience provided clues on how to integrate employability skills by rethinking the design of their courses focusing on the use of active learning, the definition of learning outcomes and an assessment no longer based on the student’s ability to memorise, as testified by one of them “*This experience will be integrated into the course preparations, specifically in the selection of the skills, how they will be put into actual practice (…) and how they will be reflected in the evidence*”.

#### Employability enhancement

The lecturers acknowledged the potential of the micro-credentialing methodology to foster employability. Most of them agreed that the badges linked to the selected evidence allow students to showcase their value as future employees: “*The EPICA ePortfolio has been proven to definitely increase the visibility of our students’ employability skills to employers, after having gone through a digital-based review and approval process by our teachers*”. Great importance was attributed by lecturers to the authenticity of the evidence from students’ lived professional or work-related experiences as they provide substantive information to employers about the students’ performance and application of knowledge and know-how to real situations and problems. Besides this, the contextualisation of the evidence with further explanation was also considered as an added value as it helps employers develop a comprehensive understanding of the presented experiences. Finally, the ePortfolio was also perceived to be an optimal tool for students to demonstrate their skills and for employers to more reliably appraise student profiles.

In addition to this, the communication between academia, employers and students encouraged by the methodology was also seen by lecturers as a means to discover more about the demands of the labour market and therefore to redesign the curriculum: “My participation in *EPICA project has helped me understand in a broader perspective what it entails to make graduates competent enough to become employable as well as create employment by themselves*”.

Regarding the visibility of skills, lecturers felt enthusiastic with the idea that students show their achievements and productions to employers or other people, like potential clients. One of the lecturers expressed: “*I encouraged students to set their profiles to a public setting. They are proud and full of esteem for their work, so they want to share it*”. Moreover, they stated that most students expressed eagerness to present their profiles to others as they were proud of the micro-credentials earned and their achievements. However, the lecturers expressed the need to give the student control over their own ePortfolios and over what they share and with whom.

#### Methodology uptake

Despite some difficulties experienced during the pilot due to Covid-19, lecturers positively valued the overall experience and expressed their willingness to integrate the micro-credentialing methodology into their teaching practices (87.5%, n = 7). However, they highlighted the need for wide adoption of a competency-based approach and for transformation of the curriculum to close the gap between graduate readiness and labour market expectations: “*My participation made me realize that the University curriculum needs transformation in order to prepare* students so that they are competent for the labour market at the time they graduate”. They also recommended extending the implementation of the micro-credentialing process to other academic programs within the university to reach a larger audience and benefit all university students.

### Student perspective

#### Pedagogical principles

**Fig. 4:**
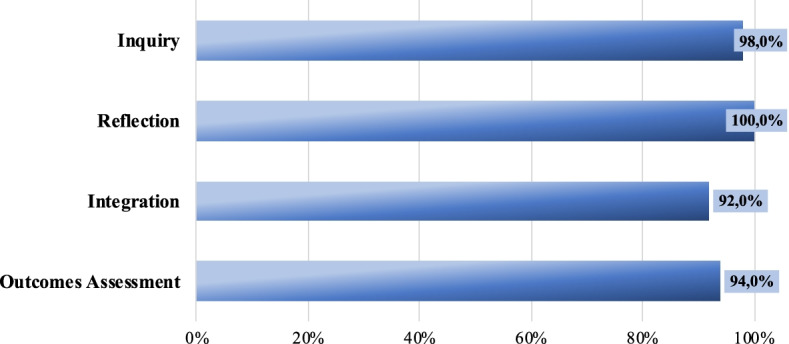
Student perceptions of pedagogical principles fostered by the ESMC. Total students: 50. Percentage of agreement based on a 1–7 scale (1 = Strongly disagree and 7 = Strongly agree) (Source: Authors. EPICA Initiative.)

Students have an even better perception than lecturers of the pedagogical dimensions of the micro-credentialing process (see Fig. 4). They also presented greater homogeneity in their opinions: inquiry (98%, n = 49 students), reflection (100%) and integration (92%, n = 46) present similar scores.

The qualitative analysis revealed that the micro-credentialing of their employability skills helped them develop a new perspective regarding their educational journey. Some students explained that identifying relevant situations and evidence demonstrating their skills raised their awareness of their usefulness within and outside their academic contexts. The formative feedback was perceived as essential to foster reflection on students’ own achievements and hence on their level of performance, progress over time and aspects to improve: “*Due to the feedback I got from my teachers and the employer about my evidence in the ePortfolio, I realized that there are some aspects that I needed to improve in my professional development*”. However, the students commented that the production of the reflective narrative linking situations and evidence to provide an integrated account of their skills proficiency was a challenging task. They appreciated the availability of the assessment rubrics at all times as a conceptual support tool for this purpose, but also as an element that contributed to the development of their self-assessment skills. Similarly, they reported that the STAR method encouraged them to identify connections between skills, tasks, actions and the results attained, improving their awareness of the skills developed in a variety of situations.

Most of the students also stated that the integrative process not only helped develop their awareness but also improved their skills (e.g. writing and editing, self-assessment, digital and analytical skills, self-presentation, self-directed learning): “*I’ve gained not only writing and editing skills, but also I’ve learned how to express myself, improving myself-esteem and confidence*”. Some students commented that they increasingly improved their performance by repeating the same process for the demonstration of each skill. The exercise of communicating the skills first to lecturers and then to employers also helped students to better understand what each target group was expecting from them.

Students also agreed with lecturers that the most highly valued aspect is the outcomes assessment (94%, n = 47) since it provided them with a clear view of their skills development: “*it is an appropriate strategy to assess the level of development of my skills in a progressive way and throughout the whole program*”.

#### Employability enhancement

The micro-credentialing process stands out among students for its potential to enhance employability. Some of them concurred that the badges awarded by the lecturers make skills not explicitly mentioned in the curriculum visible along with their level of development, and that this increases job opportunities. In addition, this process entails linking academic and professional sectors and hence putting recruiters in contact with possible candidates. This connection along with the option to interact with employers and receive their appraisal is perceived as an opportunity to understand what they are expecting and, therefore, to fine-tune the way they present their profiles.

According to most of the students, the process also provided them with the opportunity to showcase the skills developed through curricular or extracurricular activities and to connect them with real evidence and certificates demonstrating their achievements. The availability of this information makes the process behind the badge transparent for employers who have to make hiring decisions and increases trust and confidence among stakeholders.

Moreover, digital badges and the ePortfolio, by making skills visible, also provide added value to the diploma that could increase opportunities to find a job: “I think that the use of the *ePortfolio adds value to my diploma as I attain more skills and also present real up-to-date evidence that never* leaves questions hanging about the truth and in this way, this adds more employability opportunities on my scale”. Some of the students also claimed that the ePortfolio is a powerful tool to be used together with the CV as it provides the job applicant with an advantage over other candidates. The potential to enhance employability is also attributed to the use of the STAR method, which is perceived as a useful strategy to improve performance in job interviews.

Besides this, most of the students stated that they felt comfortable sharing digital badges and the related evidence with others as this exercise also increases their visibility. The majority of them shared their ePortfolio with lecturers, tutors and employers but also with colleagues, family and friends. Conversely, a few of them, while comfortable with showcasing badges, prefer to limit their sharing of their ePortfolios and evidence to lecturers, and are very cautious about what they show to others.

#### Methodology uptake

Students expressed positive attitudes regarding the micro-credentialing process and the ePortfolio and stated their intention to use them in the future (92%, n = 46).

Among the benefits they perceived is the opportunity to develop employability skills relevant to the workplace and to complement the traditional curriculum vitae (CV) with micro-credentials. However, they pointed to two additional aspects that affect successful adoption of the methodology: wider use in different programs and courses and ePortfolio ownership. Student control over their ePortfolios once their studies are finished will play a significant role in ensuring their badges remain linked to the supporting evidence and in helping them build their lifelong learning and career on a centralized platform. Likewise, some students proposed that in order to capture a comprehensive overview of the skills to be showcased to employers, their assessment should be applied throughout the whole programme.

Additionally, the students stated that they would appreciate it if employers were involved earlier in the programme as the interaction with them is key to improve their performance: “*I am of the view that employers should be in the system from the beginning of the program so as to enable students to be guided from the very beginning and improve their confidence*”.

Cooperation with peers should also be enhanced. Another suggestion that emerged that would facilitate uptake of the solution is to involve students who already know the micro-credentialing process in the training of their peers. A few students also revealed their intention to keep using it in the future due to the opportunity it offers to work from home due to the relatively low bandwidth needed. For this reason, the implementation of this methodology should be recommended to other African universities, especially after the pandemic which forced students to move online all their activities.

### Employer perspective

#### Pedagogical principles

**Fig. 5:**
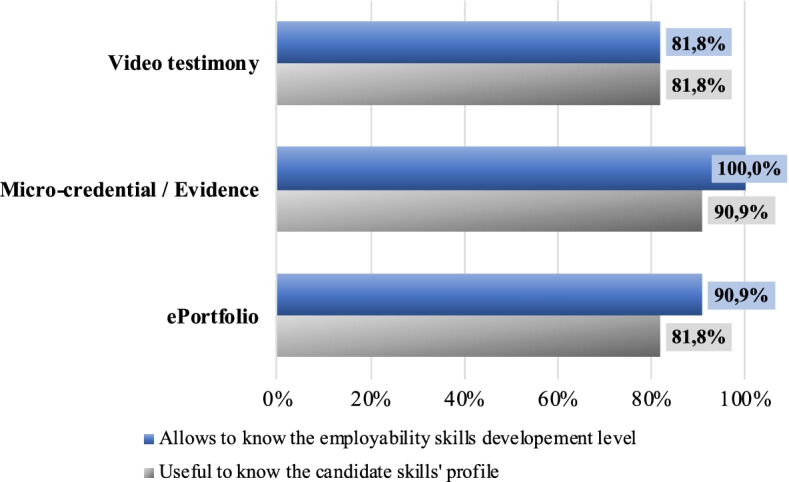
Employer perceptions of students’ skills based on the artefacts presented. Total employers: 11. Percentage of agreement based on a 1–7 scale (1 = Strongly disagree and 7 = Strongly agree) (Source: Authors. EPICA Initiative.)

**Fig. 6:**
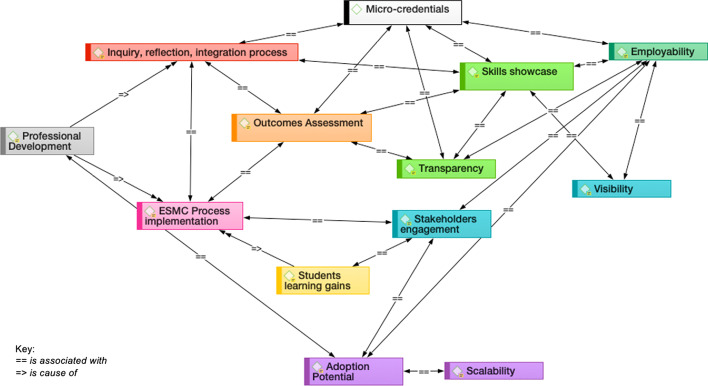
Network of the semantic linkages between the codes emerging from the lecturer data

**Fig. 7:**
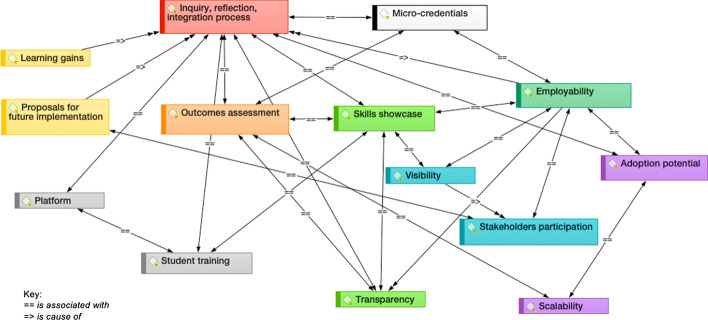
Network of the semantic linkages between codes emerging from the student data

**Fig. 8:**
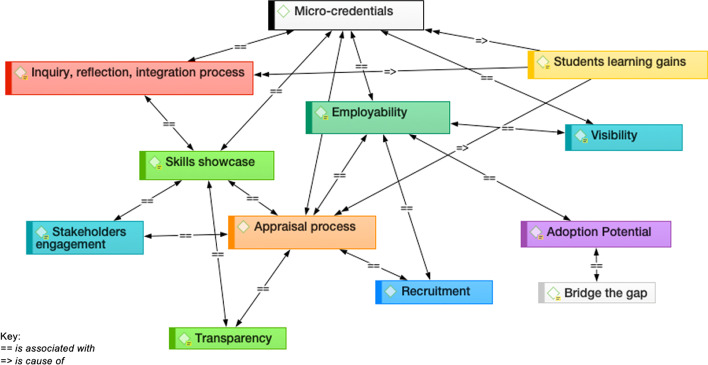
Network of the semantic linkages between codes emerging from the employer data

The overall employers’ perception of the students ePortfolio, understood as an intentionally organized collection of artefacts, showed that the ways that students expressed themselves and presented their profiles were useful both for getting to know the candidate profile (81.8%, n = 9 employers) and for verifying how skills had been developed (81.8%, n = 9). Asked also about the linking of badges to supporting evidence and the video testimony, the results also reflected high scores (see Fig. 5) in line with the overall opinion of the ePortfolio, thus reinforcing the relevance of each element in building a trustworthy portrait of the candidates’ capacities (90.9%, n = 10).

The qualitative analysis provides further information regarding employer perceptions of the experience which is seen as an innovation that enables students not only to learn but also to better prepare themselves for the labour market. Through the appraisal of students’ profiles, they manifested that the students presented significant situations revealing their capacity to perform complex tasks and that the evidence was clearly explained providing credibility to the earned micro-credentials. In addition, some of them saw the experience as an opportunity for students to improve their network of contacts, reflect on a personal development plan and career goals, and enhance the skills required by the labour market: “*It has been a good and inspiring experience for the students, in a way that it has given room to the learners to build their employability skills in communication and interpersonal skills, teamwork and problem-solving skills and with this, I believe the students can be in a position to be prepared for* the job market and also equipped with the necessary skills for it”.

Some employers mentioned that they had been pleasantly impressed by the students’ engagement in the process, their great sense of responsibility in the presentation of the contents and their enthusiasm for the feedback received.

#### Employability enhancement

Most employers consider that the micro-credential process and the ePortfolio provide new employability opportunities for graduates. The award of badges is perceived as a viable solution for showcasing skills that help differentiate and identify potential best graduates for a job. Some of them commented that the badges and the attached evidence provide a clear view of the candidate skills that is due also to the availability of rich information that complements what is reported in a traditional curriculum vitae: “*the ePortfolio provides practical evidence that supports students’ resumes/CV*”. Moreover, some of them underlined that it meets their need to see more than academic achievements reported in transcripts and resumées. Through the ePortfolio they can access a broad range of evidence that links university learning with extracurricular and work-related experiences that are of particular value in hiring processes. This also increases the reliability of the process as “*the recruitment is based on verifiable information about the candidates*”.

Most of the employers also agree that digital badges and the ePortfolio provide some advantages in the selection process over other methods. They make it easier to identify the strengths of a candidate and thus easily match organizational goals and job requirements with the graduate’s skills, qualifications, talents and personal interests. To sum up, employers recognise the potential of the EPICA solution to support job interviews and to simplify the recruitment of new candidates: “I believe this is the best system and it should be adopted and implemented because it clearly illustrates someone’s skills. All the students’ *ePortfolios I have appraised clearly illustrated their problem-solving, communication, and teamwork skills*”. According to them, the use of the STAR method also contributes to the capture of the skills of a job seeker as applied to a real context.

Additionally, some of them acknowledged the benefit of the digitalization of achievements and credentials. Making relevant information digitally available enhances graduates’ visibility and provides opportunities for students to be noticed by companies looking for candidates for a given position.

#### Methodology uptake

Most employers saw a benefit in the use of badges attached to evidence within an ePortfolio as part of the hiring processes: 81.1% said they would use it always or frequently. Although this is only a picture of employers who were inclined to participate in the pilot, their comments on the subject provide insights into their view of the EPICA solution. Their participation in the pilot was seen as having a positive impact on their efficiency in terms of productivity and the time spent on exploration of students’ profiles, and their effectiveness, due to improved performance in the identification of best candidates based on a clear understanding of their characteristics and documented previous experience. The easy access to rich profiles through digital makes it possible to foresee adoption: “(…) with the *ePortfolio* available online employers can find online students’ credentials for a particular position more easily than with a traditional CV”.

## Discussion

The analysis of each stakeholder perspective provides substantive information on the implementation of the ESMC methodology and how it can lead to curricular transformation and provide students with increased opportunities for employability. This section presents a general overview looking at how this pedagogical innovation raises students’ awareness of their actual capacities, and improves the recognition of these achievements on the basis of a formal assessment procedure, and their acknowledgement by employers.

### EMSC as a pedagogical innovation that supports the transition to the workplace

This pilot experience shows the ESMC methodology to be a promising approach for supporting students in their transition from academia to the workplace. The employability skills micro-credentialing methodology and the involvement of employers in the students’ academic journey are perceived in general as a challenging endeavour that entails rethinking of educational practices, academic curricula, and lecturers’ professional development.

With regard to the application of the ESMC methodology, the lecturers identified three main actions that challenged their practice, the first being the focus on employability skills, the second a special attention to a program view where their course is closely interlinked with other courses, and the third, the integrated approach connecting the students’ educational experiences within and outside the curriculum. This experience also enabled them to broaden their understanding of the relationship between HE and the business sector.

The students reported similar opinions in relation to the methodology that enabled them to create connections between courses and non-curricular experiences, and identify value, as is the intention of the pedagogical dimension of the Catalyst Framework (Eynon & Gambino, 2017), in terms of the mastery of their employability skills. This inquiry and reflection process, and the effort in shaping the way they introduce themselves and present their profiles to specific targets (Tomasson & Lithgow, 2018), increased their awareness of what their expectations are, and enhanced their self-esteem and self-confidence in dealing with the challenges of the professional world.

The lecturers also pointed out a shift in the assessment practices implemented during the pilot. This change was mainly driven by the fact that the students’ achievements were being considered across the programme and by the use of common criteria for the assessment of the learning outcomes. In line with Oliver (2019), both lecturers and employers emphasized the positive role of continuous evaluation, feedback, and interaction with students, although they also underline that this approach could be challenging when dealing with a large number of students. The feedback received from lecturers and employers is also perceived by the students as especially useful to improve their ability to identify and communicate their skills to specific target groups.

### EMSC fostering employability skills awareness

The students emphasized that the articulation of their employability skills has improved their awareness regarding key moments throughout the curriculum and other contexts where they have been confronted with situations requiring the use of their skills. They also highlighted that the interaction with lecturers and particularly with employers helped them identify current expectations in the labour market more clearly and increased their understanding of how employers might appraise their profile as highlighted by Tomasson Goodwin et al. (2019). This strategic knowledge is perceived as especially useful when preparing for job applications, particularly in tailoring their ePortfolio and in developing bold arguments regarding their readiness for work. Their awareness of their skills and their increasing ability to showcase them using the STAR method aligns with the postulate that the skills gap is best characterised as a ‘skills-articulation gap’ (Watkins & McKeown, 2018). The ESMC methodology, besides accrediting employability skills, also properly scaffolds students in closing this skills articulation gap and in gaining a deeper understanding of their readiness for the workplace.

The lecturers recognized that through their intervention, and the examination of employer participation, they were able to identify key curriculum changes to support students’ awareness, mainly related to the scaffolding of reflective learning and the provision of multiple opportunities for articulation.

All three stakeholders agreed on the capacity of the methodology to support learners in the identification of personal strengths and the development of a career plan.

### Micro-credentials enhancing skills visibility*,* transparency and trustability

The fact that badges provide direct access to supporting evidence through the ePortfolio is in line with initiatives focused on enhancing learners’ records (Green & Parnell, 2017) and ensuring increased visibility, transparency and trustability of the recognition process. Although this perception was shared by all participants, it was especially emphasized by employers who saw concrete advantages in the recruitment process over more traditional methods. These findings fit with the results reported by Gallagher (2018) which highlight that skills-based hiring is gaining significant interest and momentum among HR leaders. In particular, employers referred to the video testimony where they can “see and hear”' students introducing themselves, and to the clear and systematic way in which students present their awarded skills and evidence. They also pointed to the benefit of going online, facilitating access to a digital set of structured information and documentation reflecting what the student knows and can do, as advocated by Milligan and Kennedy (2017). The organized and easily navigable collection of evidence provides greater depth helping employers see what the students’ full potential could be and assists in matching the candidate profile to the job requirements.

### Extended certification, CV and employability opportunities

The students pointed out that the badges awarded enrich their current CV providing additional relevant information regarding their capacities and, in combination with the evidence stored in the ePortfolio, help them distinguish themselves from other candidates. They can be used in addition to the traditional diploma and thus increase employment opportunities. Employers, in turn, underlined that the micro-credentials provided significant additional information on skills and experiences that are not shown on academic transcripts or traditional CVs and which are highly relevant and required when deciding on a job applicant, as pointed out by Braxton et al. (2019) and Kato et al. (2020). They add that the use of micro-credentials displayed in the form of digital badges through the ePortfolio could also modernize their hiring processes, reducing the time spent by employers in reviewing candidates’ profiles and the multiple rounds of interviews. These results suggest that the dissatisfaction of the marketplace with traditional credentials might be, at least in part, mitigated by the use of the micro-credentialing system.

### ESMC methodology future implementation

From a different angle, the participants’ views and interest in the adoption of the micro-credentialing methodology provide additional information regarding its potential. The lecturers expressed their willingness to integrate the methodology into their teaching practices and to consider the pedagogical principles in the design of their courses. Similarly, students expressed their intention to keep using the EPICA solution. Future adoption, however, will require further transformation toward a more focused and explicit integration of employability skills throughout the curriculum. The lecturers concurred that successful implementation should be accompanied by significant learning-centred institutional change (Tomasson Goodwin & Lithgow, 2018) involving the redesign of the curriculum at all levels, including methodologies focused on skills development, the design of authentic learning experiences, including internships, externships and similar, and innovation in teaching practices relating to the facilitation of learning and assessment.

The students, for their part, emphasized that ownership and control over their ePortfolio beyond the end of their period of study is pivotal to encouraging their use. They pointed to new opportunities after graduation for updating their profiles and making use of digital badges in other contexts, such as embedding them in professional social networks while at the same time ensuring continued access to the related supporting evidence and experiences. Employers were also keen on using the ePortfolio, and particularly pointed to micro-credentials as trustable achievements endorsed by universities, and backed up by transparent recognition processes. As also stated by Oliver (2019), trust is a crucial part of winning stakeholders’ confidence. Employers also pointed to the benefits of the EPICA solution over other conventional hiring processes which they mainly located in a more efficient mechanism for identifying potential candidates based on tangible and clear information about the students’ experiences.

## Conclusion

Employability skills are of concern to HEIs and the business sector which questions graduates’ readiness to enter the labour market. This research presents the development and testing of the ESMC methodology for the assessment and recognition of employability skills through the implementation of an ePortfolio as a transition tool. The methodology is centred on the micro-credentialing process, providing university recognition of students’ employability skills and building external trust in students' full capacities on the basis of a transparent procedure and access to rich, concrete and multiple evidence.

The findings point to a conceptual change influencing the teaching practices of the lecturers taking part in the pilot, entailing mainly the integration of an ePortfolio strategy supporting outcomes-based assessment and the issuing of badges. Results also show that students have increased awareness of their own employability skills and of the expectations of the labour market. They became more competent in identifying their strengths and gained confidence by receiving formal university recognition through micro-credentials. Moreover, they improved their communication skills by developing academic and experiential accounts, contributing to the projection of a professional digital identity that may bring new opportunities for employability. The employers, for their part, valued the students’ presentation through the ePortfolio and the university-endorsed badges as access points to well organized and contextualized evidence. This opportunity, besides facilitating the match between candidates’ profiles and job requirements, is also a positive way of making the recognition process more transparent and trustable.

Future studies should benefit from reinforcements in curriculum design focused on employability, implementing the micro-credentialing methodology throughout the entire duration of the academic program and closely involving the business and administration sectors. A more transversal, but also progressive and sustained approach could improve not only students’ awareness but also promote further development of their skills.

## Data Availability

The data generated and analysed during this study are included in this published article and its additional information files. Additional material can be shared upon reasonable request.

## Appendix

See Table 1 and Figs. 6, 7 and 8.

## Abbreviations

CV: Curriculum vitae
EPSPI: Electronic Portfolio Student Perspective Instrument
ESMC: Employability skills micro-credentialing
H2020: Horizon 2020
HE: Higher education
HEI: Higher education institution
ILO: International Labour Organization
STAR: Situation, Task, Action, Results method
TESCEA: Transforming Employability for Social Change in East Africa
